# Estrogen Deficiency and the Origin of Obesity during Menopause

**DOI:** 10.1155/2014/757461

**Published:** 2014-03-06

**Authors:** Fernando Lizcano, Guillermo Guzmán

**Affiliations:** ^1^Biomedical Research Center, Universidad de La Sabana (CIBUS), km 7, Autopista Norte de Bogota, Chia, Colombia; ^2^Fundacion Cardio-Infantil Instituto de Cardiologia, Bogota, Colombia

## Abstract

Sex hormones strongly influence body fat distribution and adipocyte differentiation. Estrogens and testosterone differentially affect adipocyte physiology, but the importance of estrogens in the development of metabolic diseases during menopause is disputed. Estrogens and estrogen receptors regulate various aspects of glucose and lipid metabolism. Disturbances of this metabolic signal lead to the development of metabolic syndrome and a higher cardiovascular risk in women. The absence of estrogens is a clue factor in the onset of cardiovascular disease during the menopausal period, which is characterized by lipid profile variations and predominant abdominal fat accumulation. However, influence of the absence of these hormones and its relationship to higher obesity in women during menopause are not clear. This systematic review discusses of the role of estrogens and estrogen receptors in adipocyte differentiation, and its control by the central nervous systemn and the possible role of estrogen-like compounds and endocrine disruptors chemicals are discussed. Finally, the interaction between the decrease in estrogen secretion and the prevalence of obesity in menopausal women is examined. We will consider if the absence of estrogens have a significant effect of obesity in menopausal women.

## 1. Introduction

Obesity and obesity-related disorders such as diabetes mellitus type 2 (DM type 2), cardiovascular disease, and hypertension are worldwide epidemics with a greater percentage of increase in developing countries [1–3]. Many genetic and epigenetic factors determine the pathophysiology of body fat accumulation [4, 5]. The majority of these factors can be classified into different categories [6–9] such as (1) factors responsible for the hormonal regulation of appetite and satiety; (2) factors that regulate body glucose levels [10–12]; (3) regulators of basal metabolic rate [13, 14]; (4) factors that control the quantity, disposition, and distribution of fat cells [15, 16]; (5) modulators for the differentiation of progenitor cells [17, 18]; and (6) those factors that determine adipocyte cell lineage [19, 20]. Adipocytes may also regulate the production of cytokines that control the satiety and hunger centers in the central nervous system and modulate energy expenditure in other tissues [21–23].

The increases in overweight and obesity in menopausal women are important public health concerns [24, 25]. The prevalence of obesity, which is closely associated with cardiovascular risk, increases significantly in American women after they reach age 40; the prevalence reaches 65% between 40 and 59 years and 73.8% in women over age 60 [26]. Unfortunately, there are a limited number of drugs for treatment of obesity, because the majority of new products have been recalled due to side effects [27–29].

The reasons for increasing obesity in menopausal women are not clear. Some researcher arguments that the absence of estrogens may be an important obesity-triggering factor [30]. Estrogens deficiency enhances metabolic dysfunction predisposing to DM type 2, the metabolic syndrome, and cardiovascular diseases [31, 32]. As a result of increases of life expectancy in developed countries, many women will spend the second half of their lives in a state of estrogen deficiency. Thus, the contribution of estrogen deficiency in the pathobiology of multiple chronic diseases in women is emerging as a conceivable therapeutic challenge of the 21st century. However, environmental epigenetic factors may also contribute to obesity and a cultural bias that also hinders women's efforts to combat obesity [33, 34]. Perhaps it is a combination of the aforementioned factors, but the triggers for obesity require further investigation. To address this growing problem, improved understanding of how estrogens contribute to energy balance, lipid, and glucose homeostasis promises to open a novel therapeutic applications for an increasing large segment of the female population. The potential therapeutic relevance of estrogen physiology, estrogen receptors, and the estrogen pathway will be discussed in this manuscript.

## 2. Methods 

The study design was a review of existing published original papers and reviews. We conducted this review of SSBs and health outcomes in accordance with the Preferred Reporting Items for Systematic Reviews and Meta-Analysis Statement (PRISMA) [35]. PubMed publications until Nov 30, 2013, were taken into account.

## 3. Estrogens and Estrogen Receptors in Fat Metabolism

The hormones help integrate metabolic interaction among major organs that are essential for metabolically intensive activities like reproduction and metabolic function. Sex steroids are required to regulate adipocyte metabolism and also influence the sex-specific remodeling of particular adipose depots [36, 37]. In humans, the factors that control fat distribution are partially determined by sex hormones concentrations [38]. Men, on average, have less total body fat but more central/intra-abdominal adipose tissue, whereas women tend to have more total fat that favors gluteal/femoral and subcutaneous depots [39]. Weight and fat abdominal distribution differ among women of reproductive age and menopausal women [40, 41]. The decrease in estrogen levels in menopausal women is associated with the loss of subcutaneous fat and an increase in abdominal fat [42]. The importance of estrogens in subcutaneous fat accumulation is evident; in fact estrogen hormonal therapy in men also increases the amount of subcutaneous fat [43, 44].

In humans, 17-*β*-estradiol (E2) is the most potent estrogen followed by estrone (E1) and estriol (E3) [45]. The expression of genes that encode the enzymes in estrogen synthetic pathway such as aromatase and reductive 17*β*-hydroxysteroid dehydrogenases (17*β*-HSD) is critical for E2 formation [46]. Protein products of several genes with overlapping functions may confer reductive 17*β*-HSD activities in peripheral tissues [47].

Estrogens function is mediated by nuclear receptors that are transcription factors that belong to the superfamily of nuclear receptors. Two types of estrogen receptors (ERs) have been identified, the alpha (ER*α*) and beta (ER*β*) receptors [48, 49]. The classical genomic action mechanism of ER action typically occurs within hours, leading to activation or repression of target genes. In this classical signaling pathway, ligand activated ER dissociates from its chaperone heat-shock protein and binds as a dimer directly to an estrogen response element (ERE) in the promoter of target genes [50–52], although it was considered that the action of E2 was subject to an action in gene expression regulation. Recently, there is increasing evidence of nonnuclear cytosolic or plasma membrane-associate receptors that mediate nongenomic and rapid effects of several steroid hormones [53–55]. In this manner, the traditional estrogen nuclear receptors have been found to function outside of the nucleus to direct nongenomic effects [56].

Several mechanisms of membrane-signaling activation can explain rapid responses to E2. These rapid actions include activation of kinase, phosphatase, and phospholipase that can mediate calcium-dependent signaling and can mediate downstream nongenomic physiological responses, such as effects on cell cycle, cell survival, and energy metabolism [57, 58].

Human subcutaneous and visceral adipose tissues express both ER*α* and ER*β* [59, 60], whereas only ER*α* mRNA has been identified in Brown adipose tissue [61, 62]. ER*α* plays a major role in the activity of adipocytes and sexual dimorphism of fat distribution. Female and male mice that lack ER*α* have central obesity, have severe insulin resistance, and are diabetic [63–65]. Although not all studies are in agreement, polymorphism of ER*α* in humans have been associated with risk factors for cardiovascular diseases [66].

**Figure 1:**
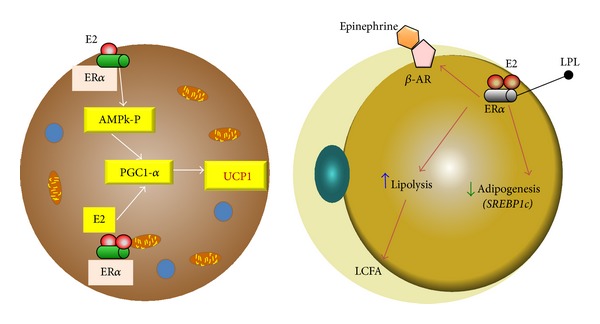
Estrogen in the fat cell. (a) In brown adipocyte cell the ER alpha receptor can increase the expression of UCP1 by increasing PGC1alpha coactivator through AMPk and by a direct effect on the receptor coactivator. (b) In white adipocyte ER alpha receptor activation by estrogen reduces lipoprotein lipase and increases beta-adrenergic receptor activity. UCP1: uncoupling protein 1; PGC1alpha: peroxisome proliferative activated receptor gamma coactivator 1 alpha; ER: estrogen receptor; AMPk: AMP-activated protein kinase. LPL: lipoprotein lipase; *β*-AR: adrenergic receptor beta.

Lipolysis in humans is controlled primarily by the action of *β*-adrenergic receptors (lipolytic) and *α*2A-adrenergic receptors (antilipolytic) [67]. Estrogen seems to promote and maintain the typical female type of fat distribution that is characterized by accumulation of adipose tissue, especially in the subcutaneous fat depot, with only modest accumulation of intra-abdominal adipose tissue [68]. Estradiol directly increases the number of antilipolytic *α*2A-adrenergic receptors in subcutaneous adipocytes [69]. Visceral adipocytes exhibit a high *α*2A/*β* ratio, and these cells are stimulated by epinephrine; in contrast, no effect of estrogen on *α*2A-adrenergic receptor mRNA expression was observed in adipocytes from the intra-abdominal fat depot [70]. However, it is important to highlight that the effects of estrogens differs on the route of administration and the lipolytic influence of estrogens on fat accumulation affects specific regions of the body [71–73]. E2 may also increase beta adrenoreceptor expression through ER*α* [74]. These results provide a mechanism insight for the effect of E2 on the maintenance of fat distribution with an increased use of lipids as energy source, which partially promotes fat reduction in abdominal fat. This effect occurs via the facilitation of fat oxidation in the muscle by the inhibition of lipogenesis in the liver and muscle through the regulation of peroxisome proliferator-activated receptor *γ* (PPAR*γ*) and an increase in LPL expression [75–77]. E2 also increases muscle oxidative capacity by means of the regulation of acyl-CoA oxidase and uncoupling proteins (UCP2-UCP3), which enhances fatty acid uptake without lipid accumulation [78, 79]. Therefore, E2 improves fat oxidation through the phosphorylation of AMP-kinase (AMPK) in muscle and myotubes in culture [80, 81] and malonyl-CoA inactivation by increasing the affinity of carnitine palmitoyltransferase [82] (Figure 1).

## 4. Estrogens Control of Central Nucleus of Appetite and Satiety 

**Figure 2:**
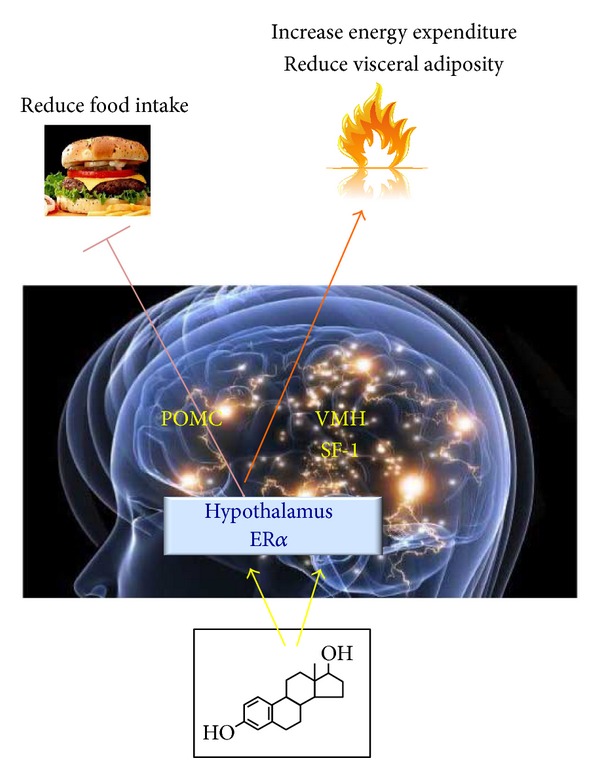
Estrogen hypothalamic control of obesity. ER alpha in the brain regulates body weight in both males and females ▸ ER alpha in female SF1 neurons regulates energy expenditure and fat distribution ▸ ER alpha in female POMC neurons regulates food intake. POMC: proopiomelanocortin; SF1: steroidogenic factor-1.

The hypothalamus is an important center in the brain for the coordination of food consumption, body weight homeostasis, and energy expenditure [83–85]. Some areas of the hypothalamus, including the ventromedial (VMN), arcuate (ARC), and paraventricular (PVN) nuclei, regulate physiological events that control weight [86]. The process by which estrogens regulate the activity of the hypothalamic nuclei is complex [87, 88]. Estrogens directly and indirectly modulate the activity of molecules involved in orexigenic action, which induces an increase in food intake [89, 90]. However, estrogen receptors regulate the neuronal activity of energy homeostasis and reproductive behaviors in a different mode. While ER*α* is abundantly expressed in the rodent brain in VMN and ARC, PVN, and the medial preoptic area, ER*β* is found in the same hypothalamic nuclei, but ER*β* expression is significantly lower relative to ER*α* [88, 91]. POMC neurons within the ARC modulate food intake, energy expenditure, and reproduction. ARC POMC ER*α* mRNA levels fluctuate over the course of the estrous cycle, with the most dramatic increase on the day of proestrus, when E2 concentration is highest [92]. Estrogens directly act on POMC neurons and regulate their cellular activity. Recent findings provide additional support for the importance of ER*α* POMC neurons and the suppression of food intake. Indeed, deletion of ER in POMC neurons in mice leads to hyperphagia without directly influencing energy expenditure or adipose tissue distribution [88]. Neuropeptide Y (NPY) is a potent orexigenic that increases food intake during fasting conditions and following food consumption by acting primarily on the ARC and PVN in the hypothalamus [93]. NPY exhibits decrease orexigenic activity after exposure to estrogens. This inhibitory action is due to the estrogen modulation of NPY mRNA expression and receptor activity [93, 94]. Ghrelin peptide is produced by parietal cells in the stomach, and it regulates feeding behaviors by sensing carbohydrate and lipid levels via stimulation of the growth hormone receptor. Ghrelin production is not limited to the stomach; different parts of the brain and some areas of the hypothalamus, such as the ARC and PVN nuclei, also produce ghrelin. Ghrelin antagonizes leptin action through the activation of hypothalamic neuropeptide Y/Y1 receptor pathway, augmented NPY gene expression, and increases food intake [95–97]. Estrogen hormone replacement therapy induces a decrease or no change in ghrelin activity [98]. Melanin-concentrating hormone (MCH) promotes food consumption by acting directly on the lateral nucleus of the hypothalamus. Nerves that stimulate the MCH activity arise from the ARC nucleus and contain POMC, NPY, and Agouti-related protein (AgRP) [99–101]. The orexigenic effect of MCH is reduced in ovariectomized rats treated with estradiol [102, 103], which is likely a direct effect of the reduced affinity of the MCH receptor or the reduced expression of MCH mRNA [104, 105] (Figure 2).

## 5. Estrogen and Energy Regulation 

**Figure 3:**
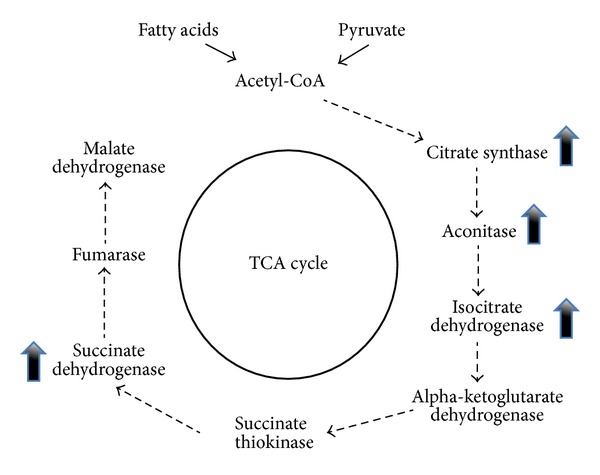
Estradiol availability affects the regulation of enzymes involved in tricarboxylic acid cycle activity. E2 enhances the glycolytic/pyruvate/acetyl-CoA pathway to generate electrons required for oxidative phosphorylation and ATP generation to sustain utilization of glucose as the primary fuel source.

E2 administered to ovariectomized (OVX) mice fed with a high-fat diet preserved improve glucose tolerance and insulin sensitivity in WT but not in ER*α*  −/− mice, suggesting that targeting of the ER*α* could represent a strategy to reduce the impact of high-fat diet induced in type 2 DM [106–108]. Insulin resistance is a central disorder in the pathogenesis of type 2 DM and also a feature observed in metabolic syndrome. Excess accumulation of adipose tissue in the central region of the body (intra-abdominal, “android,” or male-pattern obesity) correlates with increased risk of and mortality from metabolic disorders including type 2 DM [109]. As women enter menopause, there is a decline in circulating estrogen. This is accompanied by alterations in energy homeostasis that result in increases in intra-abdominal body fat. OVX rats, which are induced to exhibit obesity, regain normal weight after estrogen replacement [36, 110]. Although OVX induces a transient increase in food intake [111], hyperphagia does not fully account for changes in metabolism and development obesity after OVX [112]. Estrogens regulate glucose/energy metabolism via the direct and indirect control of the expression of enzymes that are involved in this process, such as hexokinase (HK), phosphoglucoisomerase (PGI), phosphofructokinase (PFK), aldolase (AD), glyceraldehyde 3-phosphate dehydrogenase (GAPD), phosphoglycerate kinase (PK) 6-phosphofructo 2-kinase, fructose 2,6-bisphosphatase, and glucose transporters Glut 3 and Glut 4 [79, 113–115]. Estrogens also increase the activity of several enzymes in the tricarboxylic acid cycle, including citrate synthase, mitochondrial aconitase 2, isocitrate dehydrogenase, and succinate dehydrogenase [82, 116, 117] (Figure 3).

Lipoprotein lipase (LPL) is a key-regulating enzyme for energy metabolism that breaks down plasma triglycerides into free fatty acids and glycerol. Estradiol modulates the activity of LPL wherein the promoter region contains estrogen response elements that interact with the estrogen receptor and inhibit mRNA expression in 3T3 cells and patients who are undergoing therapy with estradiol patches [118, 119]. The role of estrogens in mitochondria, which generate more than 90% of cellular ATP, must also be recognized. The mitochondria play an important role in the regulation of cell survival and apoptosis, and the respiratory chain is the primary structural and functional component that is influenced by estrogen activity [61]. The protective effect of estrogen on oxidative stress is mediated by translocation for specific enzymes from cytosol that prevent mitochondrial ADN of oxidative attack by free radicals [120].

Brown adipose (BAT) tissue is metabolically more active than white adipose tissue and its distribution changes with age. This adipose tissue is located in the neck, thorax, and major vessels in human neonates, but it is largely replaced by white adipose tissue in adults, which reaches the subcutaneous layers between muscles and the dermis, heart, kidney, and internal organs [17, 121, 122]. Brown adipose was considered absent in adult humans, but recently studies have shown that Brown adipose tissue may be stimulated in adults and might have a relevant role in the treatment of obesity [123]. Estrogens promote fat deposition after sexual maturation and alter the lipid profile. However, fat also increases in menopausal women, which suggests that estrogens play an important role in adipocyte differentiation. Experimental studies have shown that estrogens can intensify the thermogenic property of brown adipocytes, by an increase of uncoupling protein 1 (UCP1) mRNA expression [62, 124]. ER*α* is expressed in BAT tissue and mainly localized in mitochondria, which indicated that BAT mitochondria could be targeted by estrogens and pointed out the possible role of ER*α* in mitochondriogenesis [125]. Tissue estrogen sulfotransferase (EST) is a critical mediator of estrogen action. EST inhibits estrogen activity by conjugating a sulfonate group to estrogens, thereby preventing biding to estrogen receptors [126]. EST is expressed in adipose tissue and reduces adipocyte size, although an overexpression of EST in sc and visceral adipose tissue may induce insulin resistance. The role of EST in development of type 2 DM and metabolic syndrome is controversial [127, 128].

## 6. Estrogen and Adipokine Secretion

Estrogens may exert effects on several adipokines that are produced by adipocytes. Estrogen levels in premenopausal women are closely associated with leptin levels [129, 130]. Leptin may modulate energy balance in the hypothalamus by exerting an anorectic effect, and also it exhibits a lipolytic effect. Estrogen increases leptin sensitivity by controlling the expression of leptin-specific receptors [130–132].

Adiponectin is inversely associated with estrogen levels. This adipokine is involved in various inflammatory processes, endothelial function modulation, and protection against insulin resistance syndrome. Adiponectin plasma level is indirectly and negatively correlated with E2 plasma levels. Oophorectomy of adult mice increases adiponectin, which is reversed by E2 replacement [133–135].

Resistin is a hormone that is produced by adipocytes and contributes to obesity. The subcutaneous injection of estradiol benzoate reduces resistin levels in adipocytes [136].

Evidence from aromatase ArKo deficient models contributes to these observations. These mice develop a truncal obesity phenotype with increased gonadal and visceral adiposity and three times higher levels of circulating leptin without a marked increase in body weight [137]. Fat cell can produce proinflammatory adipocytokines that induce many of the complications of obesity like CD68, TNF*α*, or IL6. Administration of estrogens to ovariectomized female mice reduces significantly the mRNA of IL6, TNF*α*, and CD68. Furthermore, estrogen prevented female mice from developing liver steatosis and from becoming insulin resistant [72, 138].

## 7. Estrogen-Like Compounds and Endocrine Disruptors 

Some chemicals and plants derived compounds that may regulate the activity of estrogen receptors are potential obesogens [139]. The effect of tibolona, a synthetic substance with estrogenic activity, on body weight in postmenopausal women has been evaluated [140, 141]. One-year tibolona treatment decreases fat mass. However, tibolona combined with 17-*β*-estradiol and norethindrone acetate for 2 years does not significantly decrease fat mass [142]. The combination of hormone replacement therapy and tibolona in menopausal women increases body mass index (BMI), fat-free mass (FFM), free estrogen index (FEI), and free testosterone index (FTI), but the waist-to-hip ratio (WHR) decreases after treatment with tibolona [142]. Genistein is phytoestrogen that has similarity in structure with the human female hormone 17-*β*-estradiol, which can bind to both alpha and beta estrogen receptors and mimic the action of estrogens on target organs. Genistein is present in soy, and it is popularly used in postmenopausal women. Genistein tends to induce obesity at low doses, but higher doses increase fatty acid oxidation and reduce fat accumulation in the liver [117, 143]. However, Genistein reverses the truncal fat accumulation in postmenopausal women and ovariectomized rodent models [144, 145].

Obesity is caused by a combination of genetic and environmental factors [146]. Some xenobiotics in the environment impair the normal control of various nuclear receptors or induce an adipogenic effect. The role of these endocrine disruptors in sexual behavior, menopause, and some gonadal diseases has been examined due to their modulation of estrogen receptor activity. Numerous chemicals and plants derived compounds, such as bisphenol A (BPA), phthalates, and heavy metals, exhibit estrogenic activity [147–150]. Many endocrine disruptors may affect the transcriptional activity of nuclear receptors by changing the action of competitively binding with ligand biding domain, which may modify coactivator activity and dissociate corepressors that reduce deacetylases action. Some endocrine disruptors may also modify DNA methylation in the regulatory region of specific genes. Furthermore, some of them may activate the phosphorylation of proteins [151, 152].

**Figure 4:**
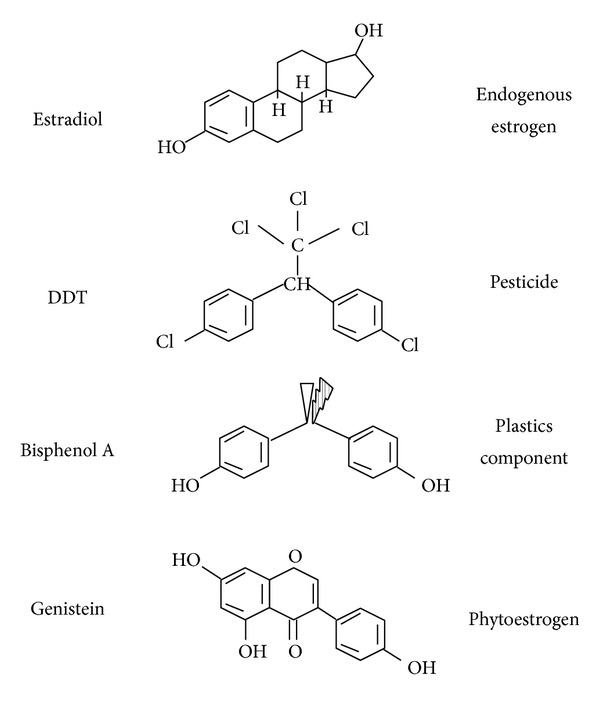
Components with estrogens effects. Estrogen and some endocrine disruptors that have an estrogenic effect. DDT is a chemical fertilizer. Bisphenol A is an organic compound used to make polycarbonate polymers and epoxy resins; Genistein is an isoflavone found in a number of plants including soy. DDT: dichlorodiphenyltrichloroethane.

Endocrine disrupters may also be involved in different estrogenic intervention processes, such as the glycolytic pathway and during the regulation of glucose transporters with compounds like BPA, 4-nonylphenol (NP), 4-octylphenol (OP), and 4-propylphenol [116, 153, 154]. Endocrine disruption using these substances also interferes with tricarboxylic acid metabolism by decreasing key enzymes in mitochondrial activity, which could be partially related to obesity (Figure 4).

In addition to these findings, many other estrogen-mediated pathways may be modulated by endocrine disruptors. Further studies are required to clarify the involvement of these chemicals.

## 8. Estrogen Therapy and Obesity

A growing body of evidence now demonstrates that estrogenic signaling can have an important role in obesity development in menopausal women. Menopausal women are three times more likely to develop obesity and metabolic syndrome abnormalities than premenopausal women [155]. Furthermore, estrogen/progestin based hormone replacement therapy in menopausal women has been shown to lower visceral adipose tissue, fasting serum glucose, and insulin levels [70, 156]. Estrogens also reduce the cardiovascular risk factors that increase during menopause. Therefore, estrogen therapy may exert a positive impact by reducing total cholesterol and relative LDL levels [157].

The estrogen type and route of administration appears to affect clinical outcomes. The changes in body fat distribution during menopause have led some researchers to suggest that hormone replacement therapy beneficially affects obesity in this group. To evaluate the metabolic effects of hormone replacement therapy using transdermal patches of 17-*β*-estradiol with medroxyprogesterone acetate in obese and nonobese menopausal women demonstrated greater fat loss [158]. Recent study participants in the most current WHI report were assigned to single estrogen therapy (0.625 mg/day conjugated equine estrogens) and combined estrogen/progestin therapy (0.625 mg conjugated equine estrogens plus 2.5 mg medroxyprogesterone acetate). An assessment of body composition using dual energy X-ray (DXA) absorptiometry at the six-year follow-up demonstrated a decrease in the loss of body fat mass with hormonal therapy in the first three years but not after six years [159–161]. However, the physiological form of estrogen is E2, and it is available in some oral preparations as well as all patches, creams, and gels for transdermal or percutaneous absorption. In contrast to orally administered HRT, transdermal delivery avoid first pass liver metabolism, thereby resulting in more stable serum levels without supraphysiological liver exposure [162].

A meta-analysis of over 100 randomized trials in menopausal women has analyzed the effect of HRT on components of metabolic syndrome. The authors conclude that, in women without diabetes, both oral and transdermal estrogen, with or without progestin, increase lean body mass, reduce abdominal fat, improve insulin resistance, decrease LDL/high-density-lipoprotein cholesterol ratio, and decrease blood pressure [163].

## 9. Conclusion

Hormone therapy during menopause will always be a mixed picture of benefits and risks. The data suggest that, for menopausal women of age 50 to 59 y or younger than age 60 y, the benefits of menopause hormone therapy outweigh the risks in many instances and particularly for relief of symptoms due to estrogen deficiency. Judgments about treatment require assessment of the needs in an individual patient and her potential for risk, such as breast cancer, coronary heart disease, fracture, stroke, obesity, and deep venous thrombosis. In order to reduce the obesity pandemic we consider that using menopause hormonal therapy with the lowest effective dose and for the shortest duration may be a possible coadjutant therapy. Future research should focus on identifying critical brain sites where ERs regulate body weight homeostasis and delineate the intracellular signal pathways that are required for the actions of estrogens. Moreover, understanding the genetics and epigenetics role of molecules that may play a role in estrogen activity in adipose tissue may reveal new pharmacological target for the beneficial action of estrogens. However, stringent studies in different locations around the world are essential to determine the real beneficial effect of estrogens for obesity treatment during menopause.
